# Neuroticism mediates the association between childhood abuse and the well-being of community dwelling adult volunteers

**DOI:** 10.1186/s13030-023-00282-5

**Published:** 2023-07-24

**Authors:** Yota Fujimura, Akiyoshi Shimura, Chihiro Morishita, Yu Tamada, Hajime Tanabe, Ichiro Kusumi, Takeshi Inoue

**Affiliations:** 1 Department of Psychiatry, Tokyo Medical University Hachioji Medical Center, 1193 Tatemachi, Hachioji, Tokyo, 193-0998 Japan; 2 Department of Psychiatry, Tokyo Medical University, 6-7-1 Nishishinjuku, Shinjuku-Ku, Tokyo, 160-0023 Japan; 3 Department of Clinical Human Sciences, Graduate School of Humanities and Social Sciences, Shizuoka University, 836 Ohya, Suruga-Ku, Shizuoka, 422-8529 Japan; 4 Department of Psychiatry, Hokkaido University Graduate School of Medicine, North 15, West 7, Kita-Ku, Sapporo, 060-8638 Japan

**Keywords:** Childhood abuse, Subjective well-being, Neuroticism, Mediating effect, Path analysis

## Abstract

**Background:**

Previous studies reported that the experience of maltreatment in childhood reduces subjective well-being in adulthood and that neuroticism is negatively associated with subjective well-being. However, the interrelationship between childhood maltreatment, adult life events, neuroticism, and subjective well-being has not been analyzed to date.

**Methods:**

A total of 404 adult volunteers provided responses to the following questionnaires: 1) Childhood Abuse and Trauma Scale, 2) Life Experiences Survey, 3) Neuroticism Subscale of the Shortened Eysenck Personality Questionnaire-Revised, and 4) Subjective Well-Being Inventory. The path model was used to analyze possible interrelationships among these parameters.

**Results:**

The effect of childhood abuse on subjective well-being was indirect and was mediated by neuroticism. The effect of neuroticism on the negative, but not positive, change score on the Life Experiences Survey was significant. The indirect effect of neuroticism on subjective well-being was not significant via either negative or positive change scores.

**Conclusions:**

This study demonstrated that age, subjective social status, neuroticism, and negative and positive life events were significantly associated with subjective well-being. Furthermore, using path analysis, we demonstrated the mediating role of neuroticism in the indirect effect of childhood abuse on subjective well-being.

## Background

The World Health Organization states that “health is a state of complete physical, mental, and social well-being and not merely the absence of disease or infirmity” [1]. Therefore, when aiming to be healthy, people should take care to maintain not only their physical condition but also their mental condition in a good state. Recently, it has been demonstrated that an individual’s experience of maltreatment in childhood interferes with the maintenance of mental well-being in adulthood [2, 3]. Maltreatment in childhood is associated with mental unstableness, including self-injury and suicidality [4]. The experience of maltreatment in childhood often induces mental disorders after the victims become adults, which then lead to a deterioration in their well-being [5]. Consistent with these findings, a previous study showed that childhood maltreatment worsened well-being in adulthood of adult volunteers [6].

Individuals who were exposed to maltreatment during middle childhood reportedly have more emotional dysregulation [7]. There is a complex association between childhood maltreatment and adult personality traits [8]. Exposure to psychological trauma in childhood is associated with stress reactivity, which is associated with a mixture of depressive, anxiety, and psychotic symptoms [9]. Maltreatment in childhood is known to be a risk factor for psychopathology in both childhood and adulthood, and various types of maltreatment in childhood have been suggested to be associated with the severity of psychological distress in adulthood [10]. Furthermore, childhood maltreatment in addition to inadequate parenting is associated with high neuroticism [11–13], and neuroticism is also associated with reduced well-being [14, 15].

Among the various personality traits, neuroticism is a major candidate as a predisposing factor of several mental disorders [16, 17]. The close association between neuroticism and depression has been thoroughly investigated, and it has been shown that neuroticism precedes the onset of major depression and that it increases the prevalence of major depression together with severe stressful life events, i.e., psychological stressors [18]. Our previous studies using structural equation modeling showed that the effects of childhood maltreatment on the development of depressive symptoms in adulthood upon exposure to stressful life events were mediated by increased neuroticism [11]. Other personality traits, such as affective temperament and the 7 dimensions of Temperament and Character Inventory, are correlated with neuroticism [19] and have been reported to mediate the association between childhood stress and well-being [6, 20]. Therefore, such a mediating role of personality traits between childhood maltreatment and well-being may also apply to neuroticism.

Based on the above background, we hypothesized that childhood abuse, adult life events, and neuroticism are interrelated and affect subjective well-being in adulthood. In this study, we sought to verify our hypothesis by analyzing interrelationships among the scores of Childhood Abuse and Trauma Scale (CATS), Life Experiences Survey (LES), Neuroticism Subscale of the Shortened Eysenck Personality Questionnaire-Revised (EPQ-R), and Subjective Well-Being Inventory (SUBI), using the path model.

## Methods

### Subjects

This study is a part of a larger study conducted on 853 Japanese adult volunteers from a community dwelling population [6]. Of the 853 volunteers, 404 (47.4%) completed the questionnaires (220 men and 184 women; age 20 to 81 years (42.3 ± 11.9 years [average ± standard deviation]). Four questionnaires and a questionnaire on demographic characteristics (sex, age, marital status, family members, employment status, education, past and family history of physical and psychiatric diseases, and subjective stratum identification) were distributed to the subjects. Written informed consent was obtained from all of the subjects after explaining the following: 1) participation in this research is completely voluntary, 2) refusal to participate will result in no disadvantage, and 3) the collected information will be kept strictly confidential.

### Questionnaires

#### Child Abuse and Trauma Scale (CATS)

The CATS is a scale that consists of 38 items. It has strong test–retest reliability (*r* = 0.71 to 0.91) and internal consistency (Cronbach’s *alpha* = 0.63 to 0.90) [21]. The scores significantly correlate with outcome measures, such as depression, dissociation, interpersonal difficulties, and stressful life events. Regarding each item, the participants rate the frequency of their experience of a particular abusive experience during childhood and adolescence using a scale ranging from 0 to 4 (0 = never; 4 = always). The total score of the CATS was used for analysis in this study.

The Japanese version of the CATS was developed and validated by the translation-back translation method. Permission of use and confirmation were obtained from the original developer of the CATS [22].

#### Life Experiences Survey (LES)

The LES is a scale consisting of 57 items. The respondents indicate the events that they have experienced within the past year [23], and they rate the effects and desirability of the events. More precisely, they indicate the events that they have experienced during the past year (within the previous 6 months and between the previous 7 months to 1 year), and (a) if they consider the event as a positive or negative experience, and (b) the degree that the particular event impacted their life when it happened. The events are rated on a 7-point scale, ranging from extremely positive to extremely negative. The sum of the impact ratings of the events that were considered as a positive experience is a *positive change score,* and the sum of the impact ratings of the events that were considered as a negative experience is a *negative change score*. The Japanese version of the LES was used in this study [24].

#### Neuroticism subscale of the shortened Eysenck Personality Questionnaire-Revised (EPQ-R)

Neuroticism was measured using the subscales of the shortened version of the Japanese EPQ-R [25]. Several studies have reported an association between neuroticism and major depression or depressive symptoms [18]. The validity and reliability of the shortened Japanese version of EPQ-R were confirmed in a previous study [26].

#### Subjective Well-Being Inventory (SUBI)

The SUBI is a self-report measurement that consists of 40 items to estimate subjective well-being (life satisfaction, happiness, etc.) and ill-being (anxiety, worry, deficiency in social contacts, etc.) [27]. The scores for all questions range from 1 to 3. Those with low scores can be interpreted as being in a less favorable state. Tonan et al. have confirmed the reliability and validity of the Japanese version of the SUBI [28]. In this study, total score of subjective well-being was used for the analysis.

### Data analysis

A structural equation model was designed in which childhood abuse predicted the subjective well-being scores of the SUBI.

Mplus version 8.5 (Muthén & Muthén, Los Angeles, CA, USA) was used to perform the path analysis to obtain the direct and indirect effects of all variables. Then, the robust maximum likelihood estimation method was used to analyze the model. To statistically evaluate the structural equation modeling, the indices of goodness of fit using the Comparative Fit Index (CFI) and the Root Mean Square Error of Approximation (RMSEA) were calculated. In accordance with the conventional criteria, a CFI greater than 0.95 and an RMSEA less than 0.08 were considered to suggest an acceptable fit; and a CFI greater than 0.97 and an RMSEA less than 0.05 was considered to suggest a good fit [29]. All coefficients were standardized and used for covariance structure analysis.

The student’s *t*-test was used to compare data between groups, and the Pearson correlation coefficient was calculated to analyze correlations between data. Using the forced entry method, multiple regression analysis was conducted with the subjective well-being score of the SUBI as a dependent variable, and the following 7 variables as independent variables: age, subjective social status (lowest = 10, to highest = 1), education (years), EPQ-R neuroticism score, total average scores of the CATS, and positive and negative change scores of the LES.

Statistical analyses were conducted using SPSS 28.0 J (IBM, Armonk, NY, USA) and Mplus version 8.5. the level of statistical significance was set at a *p*-value of less than 0.05.

## Results

### Demographic characteristics and the CATS, LES, EPQ-R, and SUBI well-being score

The demographic characteristics and CATS, LES, EPQ-R, and SUBI well-being scores of the 404 subjects are shown in Table 1. The correlation coefficient between each psychological scale is shown in Table 2. Age, education (years), subjective social status, EPQ-R neuroticism score, total scores of the CATS, and the positive and negative change scores of the LES were significantly associated with the subjective well-being scores of the SUBI assessed by the Pearson correlation coefficient and the Student *t*-test (Table 1).

**Table 1:** Characteristics, CATS, EPQ-R, and LES scores, and their correlation with SUBI subjective well-being scores or effects on SUBI subjective well-being scores in 404 adult volunteers

Characteristic or Measure		Value (number or mean ± SD)	Correlation with SUBI subjective well-being scores (*r*) or effect on them (mean ± SD of subjective well-being scores, *t*-test)
Age		42.3 ± 11.9	*r* = – 0.16^**^
Sex (male: female)		220: 184	Male 39.4 ± 6.6 vs female 38.8 ± 6.2, n.s. (*t*-test)
Education, years		15.2 ± 2.0	*r* = 0.16^**^
Employment status (employed: unemployed)		341: 56	Employed 39.0 ± 6.3 vs unemployed 39.6 ± 7.4, n.s. (*t*-test)
Marital status (married: unmarried)		287: 114	Married 39.3 ± 6.3 vs unmarried 38.4 ± 6.4, n.s. (*t*-test)
Number of cohabiters		1.8 ± 1.5	*r* = 0.010, n.s
Number of offspring		1.3 ± 1.2	*r* = 0.004, n.s
Comorbidity of physical disease (yes: no)		81: 319	Yes 38.2 ± 6.3 vs no 39.4 ± 6.4, n.s. (*t*-test)
1st-degree relative with psychiatric diseases (yes: no)		40: 362	Yes 37.7 ± 7.1 vs no 39.3 ± 6.3, n.s. (*t*-test)
Subjective social status score		4.9 ± 1.5	*r* = –0.21^**^
EPQ-R neuroticism score		3.6 ± 3.2	*r* = –0.37^**^
SUBI subjective well-being score		39.1 ± 6.4	
CATS total average score		0.65 ± 0.43	*r* = –0.23^**^
LES (change score)	Negative	1.65 ± 3.09	*r* = –0.17^**^
	Positive	1.67 ± 2.95	*r* = 0.15^**^

Notes: Data are presented as means ± standard deviation (SD) or numbers

*Abbreviations*: *CATS* Child Abuse and Trauma Scale, *EPQ-R* Eysenck Personality Questionnaire revised, *LES* Life Experiences Survey, *SUBI* Subjective Well-Being Inventory

*n.s*. not significant

*r* = Pearson correlation coefficient

^**^P < 0.01

**Table 2:** Pearson correlation coefficients between SUBI well-being score, CATS total average score, LES positive score, LES negative score, and EPQ-R neuroticism score

	SUBI subjective well-being score	CATS total average score	LES positive change score	LES negative change score	EPQ-R neuroticism score
SUBI subjective well-being score	1	–0.225^**^	0.151^**^	–0.166^**^	–0.384^**^
CATS total average score	–0.225^**^	1	–0.011	0.124^*^	0.359^**^
LES positive change score	0.151^**^	–0.011	1	0.135^**^	–0.041
LES negative change score	–0.166^**^	0.124^*^	0.135^**^	1	0.208^**^
EPQ-R neuroticism score	–0.384^**^	0.359^**^	–0.041	0.208^**^	1

*SUBI* Subjective Well-Being Inventory, *CATS* Child Abuse and Trauma Scale, *LES* Life Experiences Survey, *EPQ-R* Eysenck Personality Questionnaire revised

^*^P < 0.05

^**^P < 0.01

### Multiple regression analysis of the subjective well-being score of the SUBI using the forced entry method

The putative variables in Table 1 that showed significant correlations with subjective well-being score of the SUBI, as assessed by the Pearson correlation coefficient, or which had significant effects on the subjective well-being score of the SUBI, as determined by the Student *t*-test, were analyzed further by multiple-regression analysis.

The results of multiple regression analysis are shown in Table 3. The subjective well-being score of the SUBI was the dependent factor, and education (years), age, subjective social status (highest = 1 to lowest = 10), EPQ-R neuroticism score, total average scores of the CATS, and positive and negative change scores of the LES were independent factors. Age, subjective social status, EPQ-R neuroticism score, and negative and positive change scores of the LES were found to be significant predictors of the subjective well-being score of the SUBI (*F* = 16.93, *P* < 0.001, adjusted *R*^*2*^ = 0.221). The correlations of subjective well-being score of the SUBI with total average scores of the CATS, and education (years) were not significant. Multicollinearity was denied in the multiple regression analysis with the maximum value of VIFs in this model, 1.210 for CATS.

**Table 3:** Results of multiple regression analysis of SUBI subjective well-being scores

Independent factor	Beta	*P*-value	VIF
EPQ-R neuroticism score	− 0.325	< 0.001	1.205
Age	− 0.178	< 0.001	1.187
Subjective social status	–0.119	0.012	1.134
Positive change score of LES	0.109	0.018	1.069
Negative change score of LES	− 0.095	0.040	1.079
CATS total average score	–0.089	0.070	1.210
Education (years)	0.026	0.593	1.235
Adjusted *R*^2^ = 0.221			

*Abbreviations*: *CATS* Child Abuse and Trauma Scale, *EPQ-R* Eysenck Personality Questionnaire revised, *LES* Life Experiences Survey, *SUBI* Subjective Well-Being Inventory

Beta = standardized partial regression coefficient

Dependent factor: SUBI subjective well-being score

Independent factors: age, education (years), subjective social status (lowest = 10 to highest = 1), total average scores of CATS, EPQ-R neuroticism score, and negative and positive change scores of LES

### Path model analysis

**Fig. 1:**
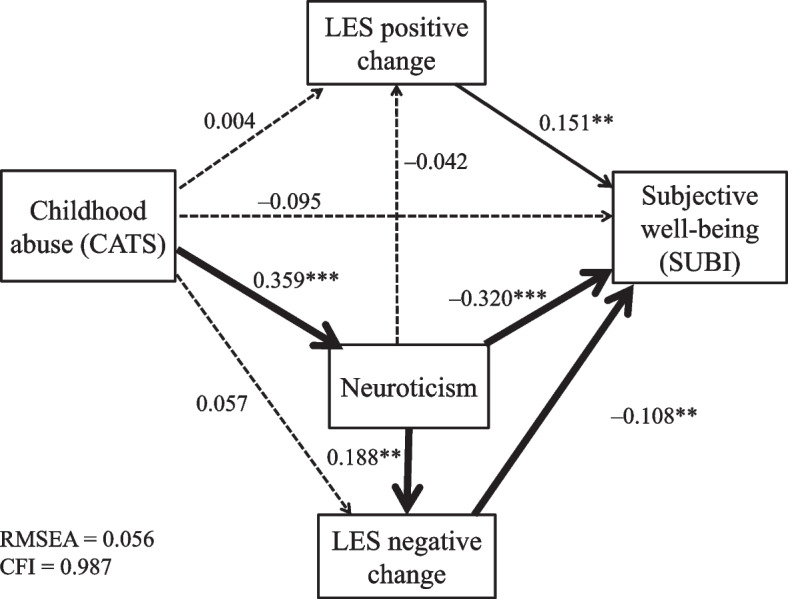
Results of covariance structure analysis in the path model, using the childhood abuse score (CATS total), neuroticism score (EPQ-R), negative and positive change scores of life events (LES), and subjective well-being score of the SUBI from 404 nonclinical adult volunteers. The arrows with solid lines represent the statistically significant paths, and those with broken lines represent the nonsignificant paths. The numbers beside the arrows show the standardized path coefficients (minimum − 1, maximum − 1). **P < 0.01, ***P < 0.001. CATS, Child Abuse and Trauma Scale; EPQ-R, Eysenck Personality Questionnaire-revised; LES, Life Experiences Survey; SUBI, Subjective Well-Being Inventory

To analyze the association between childhood abuse (CATS average total score), negative and positive change score of the LES, neuroticism (EPQ-R), and subjective well-being score of the SUBI, we built a path model based on the results of the above univariate analyses and multiple regression analyses (Fig. 1). Figure 1 shows the results of the path coefficients calculated by Mplus software.

An acceptable fit of the model (Fig. 1) was obtained based on the following criteria: CFI = 0.987 and RMSEA = 0.056. The path coefficients with solid lines in Fig. 1 were significant (*P* < 0.01–0.001). The subjective well-being score of the SUBI was significantly and directly predicted by neuroticism and the negative and positive change scores of the LES. The effect of childhood abuse on subjective the well-being score of the SUBI was indirect and was significantly mediated by neuroticism (indirect path coefficient = –0.115, *P* < 0.001). The effect of childhood abuse on the negative change score of the LES was also indirect and mediated by neuroticism (indirect path coefficient = 0.067, *P* < 0.01). However, neither the direct or indirect effect of childhood abuse on the positive change score of the LES was significant. On the other hand, the effect of neuroticism on the negative, but not positive, change score of the LES was significant. Furthermore, the indirect effect of neuroticism on the subjective well-being score of the SUBI for both the negative and positive change scores of the LES was not significant. The squared multiple correlation coefficient for subjective well-being scores of the SUBI was 0.189; i.e., this model accounts for 18.9% of the variability of subjective well-being.

## Discussion

To the best of our knowledge, this is the first study to show that childhood abuse indirectly decreases subjective well-being and that neuroticism is a mediator in this interrelationship. Using a path analysis for the data of 404 adult community dwelling volunteers, we showed that the EPQ-R neuroticism score and LES negative and positive change scores directly predict subjective well-being. Because there is a long interval between abuse in childhood and subjective well-being in adulthood, the existence of a mediator that connects these events is highly likely, and clarifying the role of the mediator will provide answers to clinical questions regarding how events that people experienced in the distant past can influence their present health.

The most important finding of this path model study is that the path analysis suggested that childhood abuse is an indirect, significant predictor of subjective well-being, although multiple regression analysis did not. If we had only performed multiple regression analysis, we might have come to the erroneous conclusion that childhood abuse is not significantly associated with subjective well-being. Our results demonstrate that the most powerful advantage of the path analysis is that it has the ability to identify indirect effects. Childhood abuse is associated with higher neuroticism [11–13]. Furthermore, neuroticism is closely associated with subjective well-being [14, 15, 30]. Even during the COVID-19 pandemic, extraversion was not significantly correlated with well-being, whereas neuroticism remained strongly associated with reduced well-being [31]. Of the 5 personality factors, neuroticism is the most strongly correlated with reduced well-being [32, 33]. This was also recently confirmed in COIVD-19-associated studies [34, 35]. Regarding the mediating effect of neuroticism, several studies demonstrated that neuroticism has a mediating role in the effects of adversity in childhood on adulthood depressive symptoms [6, 11–13, 36, 37]. Because there was a negative association between depressive symptoms and subjective well-being [6], these findings are supported by the results of the present study. Our previous studies also suggested that adverse childhood experiences such as abuse and parental bonding are perceived as being inadequate and affect subjective well-being through the mediating effect of various personality traits, such as those measured by the Temperament Evaluation of Memphis, Pisa, Paris and San Diego-autoquestionnaire version (TEMPS-A), and Temperament and Character Inventory (TCI) [6, 20]. Because subcategories of the TEMPS-A and TCI are closely associated with neuroticism [19], these previous findings are also supported by the results of the present study. Another of our previous studies suggested that neuroticism mediates the effect of childhood victimization on adulthood presenteeism in the workplace [38]. Experience of frequent work-associated bullying in adulthood was found to be associated with increased neuroticism, and the shift from a “bullied” to a “non-bullied” situation decreased neuroticism [39]. This finding may explain the mechanism of the mediating effect of neuroticism. Taken together, these previous findings and the findings of our present study indicate that both the experience of childhood trauma and traumatic events in adulthood influence subjective well-being in adulthood.

The necessity of treatment for patients who experienced child abuse is well known, and various forms of psychotherapy, such as trauma-focused cognitive behavioral therapy (TF-CBT), have been developed for such patients [40]. A clinical trial that investigated the efficacy of animal-assisted therapy (AAT) as an adjunct to TF-CBT was conducted recently, although AAT was not warranted at this time [41]. In addition to focusing on treating child abuse, we wish to focus on treating neuroticism. Because our present study suggested that neuroticism has an inhibitory mediating effect on subjective well-being in the nonclinical adult population, this knowledge may contribute to improving clients’ current subjective well-being by improving the treatment of neuroticism. Because neuroticism is a natural temperament, treating it may be considered unusual. However, opposite to theoretical conceptions of personality, recent research suggests that neuroticism is changeable over time and that it responds to treatment; i.e., neuroticism can be reduced by direct interventions [42]. Psychological treatments, such as mindfulness-based cognitive therapy, have demonstrated a significant reduction in neuroticism [43]. Another effective intervention is pharmacological intervention. Serotonergic drugs appear to induce negative effects on neuroticism, whereas noradrenergic drugs enhance extroversion [42, 44, 45]. The fact that both psychological and pharmacological interventions are effective in treating neuroticism suggests that some biopsychosocial mechanism underlies the results of the present study.

Recent progress in genomic research has shed light on this research field. A twin polygenic study suggested that genetic scores of neuroticism are associated with the risk of experiencing abuse, and this inherited genetic risk partially explains the association of neuroticism and childhood abuse [46]. Another genetic study using the genomewide association method identified genetic variants that are associated with neuroticism, depressive symptoms, and low subjective well-being [47]. Furthermore, a recent genomewide association study conducted on 329,821 individuals identified 116 independent gene variants that influence neuroticism [48]. These genetic findings may partially explain the links between childhood abuse, neuroticism, and well-being. Further biological studies are needed to clarify the mechanism underlying the mediating role of neuroticism in the effect of the experience of abuse in childhood on well-being in adulthood.

The present study has several limitations. Firstly, the sample was a nonclinical adult population. Therefore, the findings may not be directly applicable to patients with psychiatric disorders. The mediating effect of neuroticism between childhood abuse and subjective well-being should be studied prospectively in the future using a larger number of subjects, including psychiatric patients. Secondly, in this study, childhood abuse was reported retrospectively. Therefore, it relied on the subjects’ memory and self-declaration. To verify the influence of childhood abuse on subjective well-being, a prospective study of a birth cohort in which childhood abuse is objectively evaluated is needed.

## Conclusion

In this study, we demonstrated that age, subjective social status, neuroticism, and negative and positive life events were significantly associated with subjective well-being. Furthermore, using path analysis, we clarified the mediating role of neuroticism in the indirect effect of childhood abuse on subjective well-being.

## Data Availability

Detailed data are available from the corresponding author upon reasonable request.

## Abbreviations

CATS: Childhood Abuse and Trauma Scale
LES: Life Experiences Survey
EPQ-R: Neuroticism Subscale of the Shortened Eysenck Personality Questionnaire-Revised and
SUBI: Subjective Well-Being Inventory

## References

[1] World Health Organization (1946). Definition of Health in Preamble to the Constitution of the World Health Organization.

[2] Springer K, Sheridan J, Kuo D, Carnes M (2007). Long-term physical and mental health consequences of childhood physical abuse: results from a large population-based sample of men and women. Child Abuse Negl.

[3] Pitzer LM, Fingerman KL (2010). Psychosocial resources and associations between childhood physical abuse and adult well-being. J Gerontol B Psychol Sci Soc Sci.

[4] Serafini G, Canepa G, Adavastro G (2017). The relationship between childhood maltreatment and nonsuicidal self-injury: a systematic review. Front Psychiatry.

[5] Li M, D’Arcy C, Meng X (2016). Maltreatment in childhood substantially increases the risk of adult depression and anxiety in prospective cohort studies: systematic review, meta-analysis, and proportional attributable fractions. Psychol Med.

[6] Kanai Y, Takaesu Y, Nakai Y, Ichiki M, Sato M, Matsumoto Y, Ishikawa J, Ono Y, Murakoshi A, Tanabe H (2016). The influence of childhood abuse, adult life events, and affective temperaments on the well-being of the general, nonclinical adult population. Neuropsychiatr Dis Treat.

[7] Dunn EC, Nishimi K, Gomez SH, Powers A, Bradley B (2018). Developmental timing of trauma exposure and emotion dysregulation in adulthood: are there sensitive periods when trauma is most harmful?. J Affect Disord.

[8] Hengartner MP, Cohen LJ, Rodgers S, Muller M, Rossler W, Ajdacic-Gross V (2015). Association between childhood maltreatment and normal adult personality traits: exploration of an understudied field. J Pers Disord.

[9] van Nierop M, Lecei A, Myin-Germeys I, Collip D, Viechtbauer W, Jacobs N (2018). Stress reactivity links childhood trauma exposure to an admixture of depressive, anxiety, and psychosis symptoms. Psychiatry Res.

[10] Jaffee SR (2017). Child maltreatment and risk for psychopathology in childhood and adulthood. Annu Rev Clin Psychol.

[11] Ono K, Takaesu Y, Nakai Y, Shimura A, Ono Y, Murakoshi A, Matsumoto Y, Tanabe H, Kusumi I, Inoue T (2017). Associations among depressive symptoms, childhood abuse, neuroticism, and adult stressful life events in the general adult population. Neuropsychiatr Dis Treat.

[12] Ono Y, Takaesu Y, Nakai Y, Ichiki M, Masuya J, Kusumi I, Inoue T (2017). The influence of parental care and overprotection, neuroticism and adult stressful life events on depressive symptoms in the general adult population. J Affect Disord.

[13] Enns MW, Cox BJ, Larsen DK (2000). Perceptions of parental bonding and symptom severity in adults with epression: mediation by personality dimensions. Can J Psychiatry.

[14] Steel P, Schmidt J, Shultz J (2008). Refining the relationship between personality and subjective well-being. Psychol Bull.

[15] Ng W, Kang SH (2022). Predictors of well-being during the COVID-19 pandemic: The importance of financial satisfaction and neuroticism. J Community Psychol.

[16] Ormel J, Jeronimus BF, Kotov R, Riese H, Bos EH, Hankin B, Rosmalen JGM, Oldehinkel AJ (2013). Neuroticism and common mental disorders: meaning and utility of a complex relationship. Clin Psychol Rev.

[17] Malouff JM, Thorsteinsson EB, Schutte NS (2005). The relationship between the five-factor model of personality and symptoms of clinical disorders: a meta-analysis. J Psychopathol Behav Assess.

[18] Kendler KS, Kuhn J, Prescott CA (2004). The interrelationship of neuroticism, sex, and stressful life events in the prediction of episodes of major depression. Am J Psychiatry.

[19] Gonda X, Fountoulakis KN, Juhasz G, Rihmer Z, Lazary J, Laszik A, Akiskal HS, Bagdy G (2009). Association of the s allele of the 5-HTTLPR with neuroticism-related traits and temperaments in a psychiatrically healthy population. Eur Arch Psychiatry Clin Neurosci.

[20] Murakoshi A, Mitsui N, Masuya J, Fujimura Y, Higashi S, Kusumi I, Inoue T (2020). Personality traits mediate the association between perceived parental bonding and well-being in adult volunteers from the community. Biopsychosoc Med.

[21] Sanders B, Becker-Lausen E (1995). The measurement of psychological maltreatment: early data on the child abuse and trauma scale. Child Abuse Negl.

[22] Tanabe H, Ozawa S, Goto K (2010). Psychometric properties of the Japanese version of the Child Abuse and Trauma Scale (CATS).

[23] Sarason IG, Johnson JH, Siegel JM (1978). Assessing the impact of life changes: development of the life experiences survey. J Consult Clin Psychol.

[24] Nakai Y, Inoue T, Toda H (2014). The influence of childhood abuse, adult stressful life events and temperaments on depressive symptoms in the nonclinical general adult population. J Affect Disord.

[25] Eysenck S, Eysenck H (1985). A revised version of the psychoticsm scale. Person Individ Diff.

[26] Nakai Y, Inoue T, Toyomaki A, Waktsuki Y, Mitsui N, Kitaiti Y, Nakagawa S, Nakato Y, Kameyama R, Otomo Y (2015). A study of validity about Japanese version of neuroticism scores of the shortened EPQ-R.

[27] World Health Organization. Assessment of Subjective Wel-Being: The Subiective Well-Being lnventory (1992). New Delhi.

[28] Tonan K, Sonoda A, Ono Y (1995). Production of the subjective well-being inventory Japanese edition: Its reliability and validity. Jpn J Health Psychol.

[29] Schermelleh-Engel K, Moosbrugger H, Müller H (2003). Evaluating the fit of structural equation models: tests of significance and descriptive goodness-of-fit measures. Meth Psychol Res.

[30] Anglim J, Horwood S, Smillie LD, Marrero RJ, Wood JK (2020). Predicting psychological and subjective well-being from personality: a meta-analysis. Psychol Bull.

[31] Gubler DA, Makowski LM, Troche SJ, Schlegel K (2021). Loneliness and well-being during the Covid-19 pandemic: associations with personality and emotion regulation. J Happiness Stud.

[32] Isabel A, Margarida PL, Marcela M, Clau’dia F (2012). Personality and subjective well-being: what hides behind global analyses?. Soc Indic Res.

[33] Susanne B, Marlies M, Jaap JAD, Maike L (2020). Loneliness and the big five personality traits: a meta-analysis. Eur J Pers.

[34] Aschwanden D, Strickhouser JE, Sesker AA, Lee JH, Luchetti M, Stephan Y, Sutin AR, Terracciano A. Psychological and behavioural responses to coronavirus disease 2019: the role of personality. Eur J Pers. 2020; 10.1002/per.2281. 10.1002/per.2281. 10.1002/per.2281 PMC7361622 32836766

[35] Nick M, Le Vy P, Niclas K, Rauthmann JF (2021). Who is impacted? Personality predicts individual differences in psychological consequences of the COVID-19 pandemic in Germany. Soc Psychol Personal Sci.

[36] Tachi S, Asamizu M, Uchida Y, Katayama S, Naruse M, Masuya J, Ichiki M, Inoue T (2019). Victimization in childhood affects depression in adulthood via neuroticism: a path analysis study. Neuropsychiatr Dis Treat.

[37] Masuya J, Ichiki M, Morishita C, Higashiyama M, Ono M, Honyashiki M, Iwata Y, Tanabe H, Inoue T (2022). Childhood victimization and neuroticism mediate the effects of childhood abuse on adulthood depressive symptoms in volunteers. Neuropsychiatr Dis Treat.

[38] Hashimoto S, Ichiki M, Ishii Y, Morishita C, Shimura A, Kusumi I, Inoue T, Masuya J (2022). Victimization in childhood influences presenteeism in adulthood via mediation by neuroticism and perceived job stressors. Neuropsychiatr Dis Treat.

[39] Persson R, Høgh A, Grynderup MB (2016). Relationship between changes in workplace bullying status and the reporting of personality characteristics. J Occup Environ Med.

[40] Cary CE, McMillen JC (2012). The data behind the dissemination: A systematic review of trauma-focused cognitive-behavioral therapy for use with children and youth. Child Youth Serv Rev.

[41] Allen B, Shenk CE, Dreschel NE (2022). Integrating animal-assisted therapy into TF-CBT for abused youth with PTSD: a randomized controlled feasibility trial. Child Maltreat.

[42] Sauer-Zavala S, Wilner JG, Barlow DH (2017). Addressing neuroticism in psychological treatment. Personal Disord.

[43] Armstrong L, Rimes KA (2016). Mindfulness-based cognitive therapy for neuroticism (stress vulnerability): a pilot randomized study. Behav Ther.

[44] Quilty LC, Meusel LA, Bagby RM (2008). Neuroticism as a mediator of treatment response to SSRIs in major depressive disorder. J Affect Disord.

[45] Tomarken AJ, Dichter GS, Freid C, Addington S, Shelton RC (2004). Assessing the effects of bupropion SR on mood dimensions of depression. J Affect Disord.

[46] Andrew R, Karestan CK, Lori BC, Marc GW, Janet WRE, Andrea LR (2021). Polygenic risk for autism, attention-deficit hyperactivity disorder, schizophrenia, major depressive disorder, and neuroticism is associated with the experience of childhood abuse. Mol Psychiatry.

[47] Aysu O, Bart MLB, Jan-Emmanuel DN, Patrick T, Michel GN, Mark AF, Fleur WMS, Richard KL, Cornelius AR, Jaime D (2016). Genetic variants associated with subjective well-being, depressive symptoms, and neuroticism identified through genome-wide analyses. Nat Genet.

[48] Luciano M, Hagenaars SP, Davies G, Hill WD, Clarke TK, Shirali M, Harris SE, Marioni RE, Liewald DC, Fawns-Ritchie C (2018). Association analysis in over 329,000 individuals identifies 116 independent variants influencing neuroticism. Nat Genet.

